# Psoriasis and Lupus Erythematosus—Similarities and Differences between Two Autoimmune Diseases

**DOI:** 10.3390/jcm13154361

**Published:** 2024-07-25

**Authors:** Aleksandra Fijałkowska, Joanna Wojtania, Anna Woźniacka, Ewa Robak

**Affiliations:** Department of Dermatology and Venereology, Medical University of Lodz, Haller sq. 1, 90-647 Lodz, Poland; aleksandra.fijalkowska@stud.umed.lodz.pl (A.F.); joanna.wojtania@outlook.com (J.W.); anna.wozniacka@umed.lodz.pl (A.W.)

**Keywords:** lupus erythematosus, psoriasis, similarities, differences, pathogenesis, treatment, coexistence

## Abstract

Systemic lupus erythematosus (SLE) and psoriasis (Ps) are two clinically distinct diseases with different pathogenesis. However, recent studies indicate some similarities in both clinical presentation and pathogenetic mechanisms. The coexistence of both entities is very uncommon and has not been fully elucidated. Thus, it remains a diagnostic and therapeutic challenge. In fact, drugs used in SLE can induce psoriatic lesions, whereas phototherapy effective in Ps is an important factor provoking skin lesions in patients with SLE. The aim of this work is to discuss in detail the common pathogenetic elements and the therapeutic options effective in both diseases.

## 1. Introduction

As the largest organ of the human body, the skin is also one of the most significant elements of its immune system [1]. It acts as a protective barrier, separating the inside of the body from the external environment, while also mediating communication between the two. To achieve this, the skin provides adequate conditions for numerous processes that ensure the effectiveness of the immune system. However, any disruption of this balance may induce certain pathological reactions, such as inflammation and autoimmunization, which underlie various skin diseases [2]. Although the direct cause of autoimmunization is not fully elucidated, evidence suggests it may be stimulated by non-specific inflammatory responses [3]. Furthermore, exposure to certain epigenetic factors may induce related autoimmune diseases, particularly in patients with a genetic predisposition [4,5]. Unfortunately, the molecular mechanisms of such autoimmune diseases remain complex and obscure, and many are currently incurable. Nevertheless, research is ongoing to explain these processes [6].

Two inflammatory diseases with an autoimmune background are psoriasis (Ps) and systemic lupus erythematosus (SLE). Despite different skin manifestations, both diseases have an increased risk of occurrence in patients with other autoimmune diseases compared to the general population [7,8]. The two diseases tend to affect the same organs and systems, such as the skin and the osteoarticular and circulatory systems, and both conditions may co-occur with secondary metabolic disorders [9,10,11]. Ps has been reported to coexist with rheumatoid arthritis (RA), alopecia areata, celiac disease, systemic sclerosis (SSc), Crohn’s disease, Sjögren’s syndrome (Ss), vitiligo, ulcerative colitis, chronic urticaria, or giant cell arteritis [8,12,13,14,15]. In contrast, SLE tends to occur with other connective tissue diseases, such as Ss, SSc, RA, dermatomyositis/polymyositis (DM/PM), or mixed connective tissue disease. Cases of comorbid Ps and SLE are rare, with an estimated incidence of approximately 0.69% [7,11,16,17,18,19,20,21].

Psoriasis manifests in about 3% of the general population, and its first symptoms usually occur in the second decade of life; in such cases, immune dysfunction is dependent on T lymphocytes [22]. As many as 90% of cases are classed as Ps vulgaris [23]. A key factor in its development is the genetic background of the patient. The inheritance is polygenic; *PSORS1-9*, *CDKAL1*, *ZNF313*, and *PTPN22* are all known to increase the predisposition to Ps, with the *PTPN22* gene also being involved in the etiopathogenesis of SLE [24,25].

**Figure 1:**
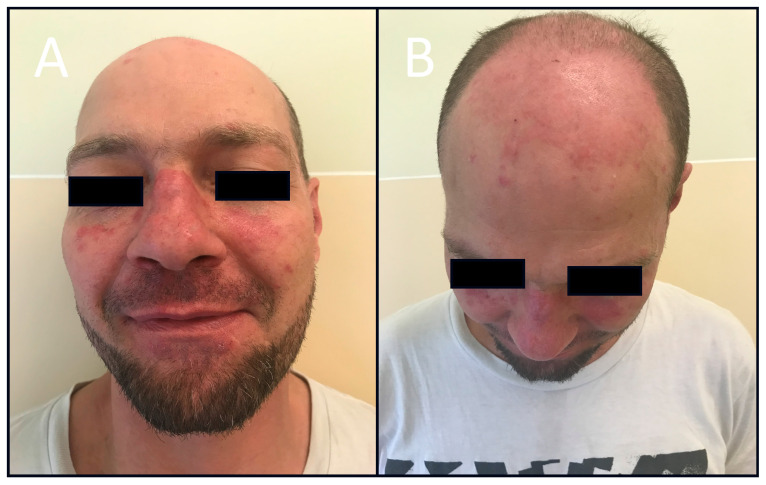
Patient with the coexistence of SLE and psoriasis. Facial erythematous and papular lesions resembling malar rash in SLE (**A**). Typical psoriatic lesions of the scalp (**B**).

SLE is a model autoimmune disease with a diverse clinical picture. It often co-occurs with involvement of the skin, kidneys, central nervous system, and hematopoietic system (Figure 1) [26]. Young women are most often affected, suggesting that female sex hormones may be involved in the pathogenesis of SLE [27,28,29,30,31,32]; no such relationship is observed in Ps [33,34,35,36]. SLE development is also believed to be primarily associated with the production of antigen-specific polyclonal antibodies: these form immune complexes that are deposited in the skin and in various organs, resulting in damage [37,38].

Ps and SLE rarely co-occur, and the relationship between these diseases remains unclear. However, it has been postulated that the two share a common molecular-level inducer that drives the development of clinical manifestations and abnormalities [39]. For many years, it was assumed that Ps was an inflammatory skin disease in which immune disorders were also present, while SLE has always been categorized as an autoimmune disease, in which inflammation was identified more as a reaction secondary to immune disorders. Furthermore, although the same drugs, such as methotrexate (MTX), cyclosporine A (CsA), and glucocorticosteroids (GCS), have been used for the systemic treatment of both, the two diseases have never been linked in terms of pathogenesis [39].

**Figure 2:**
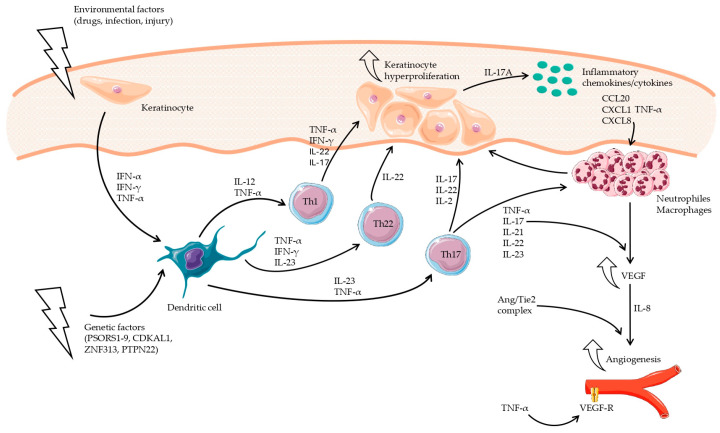
Cytokine network in the pathogenesis of psoriasis. Activated dendritic cells produce IL-23, which stimulates Th17 cells to produce inflammatory cytokines, including IL-17, which stimulates hyperproliferation of keratinocytes. Activated keratinocytes produce chemokines that enhance the migration of inflammatory cells into psoriatic lesions, which induce further keratinocyte proliferation and sustain the self-perpetuating inflammatory mechanism. IL-17 increases VEGF and IL-8 levels, which stimulate angiogenesis.

**Figure 3:**
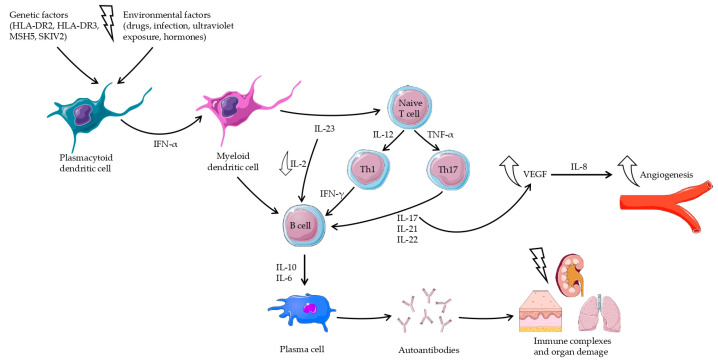
Cytokine network in the pathogenesis of systemic lupus erythematosus. IFN-α-activated myeloid dendritic cells enhance the differentiation and proliferation of naive T cells. Emerging Th1 cells produce IFN-γ, and Th17 cells produce IL-17, IL-21, and IL-22. These inflammatory cytokines stimulate proliferation of B lymphocytes, which increase plasma cell levels and the production of autoantibodies. The resulting immune complexes induce multi-organ damage.

Recent studies indicate that in Ps, specific inflammation is provoked secondary to immune dysfunction, as noted in SLE; however, in Ps, the response is dependent on Th1 and Th17 lymphocytes (Figure 2), while in SLE, it is mainly dependent on Th2 and B lymphocytes (Figure 3). The pathogenesis of SLE is still being extensively studied, and existing results indicate that the pathomechanisms are complex and dependent on multiple mechanisms. Nevertheless, in-depth studies have found that the two to share similar abnormalities in signaling pathways, in which inflammation depends on cytokines released by Th1, Th2, Th17, B lymphocytes, and chemokines and abnormal angiogenesis [12,40].

The aim of this review is to analyze the existing state of knowledge on the comorbidity of Ps and SLE with a particular emphasis on their mutual pathogenetic mechanisms and similarities in the clinical picture. A thorough understanding of the immunopathogenesis of these diseases may contribute to the design of new drugs that would provide therapy targeted at the direct cause, making a cure possible.

## 2. Autoimmune Background

Research has attempted to identify associations between SLE and Ps. Singh et al. showed that 28.8% of a study population of Ps patients had rheumatoid factor (RF) or autoantibodies present, including anti-double-stranded DNA (anti-dsDNA) antibodies. The presence of the latter is of great diagnostic and prognostic significance in SLE, as their titers are considered a marker of activity of the disease process, and their presence is more often observed in patients with renal involvement. Currently, the most important element in the diagnosis of SLE is the presence of antinuclear antibodies (ANA), and this is a condition for verification of the diagnosis based on the current ACR/EULAR 2019 classification criteria. However, not all patients presenting ANA will reveal systemic connective tissue disease [41,42]. Nevertheless, Ps patients have a significantly higher incidence of circulating ANA compared to the healthy population. Marín-Acevedo et al. confirmed the presence of ANA in 35 (44.3%) of 79 Ps patients (45 women and 34 men) and in seven healthy controls (11.5%) [43]. 

In the case of overlap between SLE and Ps, an important factor in the diagnosis is the presence of anti-Ro antibodies [39,44]. A positive correlation has been shown between the occurrence of these antibodies and photosensitivity. UV radiation increases the expression of Ro antigen on the surface of keratinocytes; therefore, high levels of serum anti-Ro antibodies can stimulate cytotoxicity and an antibody-mediated immune response [45]. This can result in damage to the basal layer of the epidermis and the appearance of lesions on the skin, especially in areas of UV exposure. Anti-Ro antibodies are most commonly detected (70–90%) in patients with subacute cutaneous lupus erythematosus (SCLE). This lupus variant is characterized by a greater incidence of psoriasis-like skin lesions and more intense photosensitivity compared to other forms of lupus [46]. 

In contrast to SLE patients, one of the most commonly used treatments in Ps patients is phototherapy. UV radiation inhibits the Th1/Th17 lymphocyte-dependent inflammatory response, induces T-cell apoptosis in the epidermis and dermis, and suppresses the activity of Langerhans cells, thus suppressing the immune response [47]. Psoriatic lesions are rarely exacerbated by exposure to the sun (5–20%), and the mechanism of this phenomenon remains unclear [48]. Nevertheless, a study conducted in 1983 showed that patients with Ps and overlapping SLE may be at increased risk of photo-induced skin eruptions due to the higher prevalence of anti-Ro antibodies. However, due to the relatively small study group of 4 patients with Ps and overlapping SLE, further research is necessary to explain this correlation [49].

## 3. Cytokines in Psoriasis 

### 3.1. IL-17/IL-23/TNF-α 

IL-17 is a pro-inflammatory cytokine produced by activated Th17 lymphocytes and, to a lesser extent, by Th1 lymphocytes. The cytokine is crucial in the pathogenesis of many autoimmune diseases, such as Ps, psoriatic arthritis (PsA), multiple sclerosis, and inflammatory bowel diseases [50,51,52,53]. The involvement of IL-17 in the acute inflammatory response has been well documented; in addition, it is believed to play a role in CRP protein synthesis, which confirm its influence in the development of chronic inflammation [54].

In Ps patients, certain drugs, infection, or stress can affect keratinocytes and stimulate the production of pro-inflammatory cytokines, i.e., interferon-α (IFN-α), interferon-γ (IFN-γ), and tumor necrosis factor-α (TNF-α). These cytokines stimulate dendritic cells (DCs) responsible for T-lymphocyte activation and influence their differentiation into Th1 and Th17 cells. These migrate into the skin, where in the presence of IL-23, Th17 releases IL-17 (A-F), IL-21, IL-22, and IL-23, while in the presence of IL-12, Th1 increases TNF-α and IFN-γ levels [12,55,56,57,58,59]. 

Overactivity of the IL-17/IL-23/TNF-α axis and overproduction of IL-12, IL-21, IL-22, and IFN-γ play important parts in the development of Ps. These initiate a self-perpetuating inflammatory mechanism, resulting in the abnormal proliferation and differentiation of keratinocytes and thus the formation of typical psoriatic lesions on the skin [59,60,61,62,63]. IL-17A also stimulates keratinocytes to produce IL-19 and the release of keratinocyte growth factor by fibroblasts. Moreover, IL-17A enhances the synthesis of inflammatory chemokines CXCL1 and CXCL8 involved in neutrophil recruitment and CCL20 production. Chemokine CCL20, also known as liver and activation-regulated chemokine (LARC) and macrophage inflammatory protein-3 (MIP3A), is a protein belonging to the CC chemokine family. It shows a strong chemotactic effect on lymphocytes and a lesser one on neutrophils [64]. Hyperreactivity of the CCL20/CCR6 axis has been shown to sustain inflammation in Ps [65,66]. 

Ps patients demonstrate high plasma levels of IL-21 and IL-22, which contribute to epidermal barrier dysfunction and promote increased epidermal cell differentiation [12,58]. Furthermore, IL-21 is also thought to stimulate the proliferation of Th17 cells through a feedback mechanism, consequently increasing IL-17 levels in the blood [58]. IL-23 promotes the differentiation of naive T cells to Th17 and their survival. It also shows a positive correlation with disease activity in the course of Ps, with the concentration increasing in skin lesions during active disease and decreasing during effective therapy [58]. However, no significant relationship has been noted between plasma IL-23 levels and the severity of the disease assessed using the Psoriasis Area and Severity Index (PASI) or with the affected body surface area (BSA) [67].

### 3.2. IL-2 

The gene encoding IL-2, which is located on chromosome 4q27, has been found to house polymorphisms that are associated with various autoimmune diseases, including SLE, Ps, RA, ulcerative colitis, diabetes, and asthma. This suggests that these diseases share a similar genetic predisposition [58,68]. IL-2 has a specific role in regulating immune processes. At high concentrations, it stimulates the proliferation and differentiation of naive T cells, while at low concentrations (low-dose IL-2), it can activate Treg cells, which are involved in the development of immune tolerance [69]. IL-2 deficiency or abnormal IL-2 activity results in reduced Treg cell count and dysfunction, which predisposes to the development of an autoimmune response [70]. The significance of IL-2 in the etiopathogenesis of Ps has not been thoroughly elucidated. However, the cytokine is produced by activated Th1 and Th17 cells, which play a key role in the pathogenesis of Ps [71].

One study on Ps attempted to restore the proper Treg/Th17 ratio using a regimen based on therapeutic low-dose IL-2. Briefly, 45 patients with Ps received 7.5 mg MTX once a week for 12 weeks. From week 13 of treatment, this was supplemented with daily low-dose IL-2 at a dose of 0.5 million international units (IU) by subcutaneous injection for two consecutive weeks, followed by a two-week break [72]. Such modified therapy was continued until week 24. After the end of treatment, significant reductions in inflammatory markers were noted in peripheral blood compared to baseline; these included leukocyte and neutrophil counts, ESR, CRP, and the neutrophil-to-lymphocyte ratio (NLR). However, lymphocyte and platelet counts as well as the platelet-to-lymphocyte ratio (PLR) did not change. A two-fold increase in the percentage of Tregs in the peripheral blood was observed together with increased concentrations of the anti-inflammatory cytokines IL-4 and IL-10 [72]. Another study by Wang et al. found low-dose IL-2 therapy to achieve similar results to immunosuppressive drugs, such as prednisone, ameliorating the course of PsA without causing side effects [73].

Despite its benefits, IL-2 is highly toxic, and its use requires an experienced medical team and close adherence to the strict qualification criteria; as such, unlike anti-IL-17 or anti-TNF, it has not yet been widely used in therapy. The most common side effect of IL-2 immunotherapy is vascular leak syndrome associated with increased blood vessel permeability and fluid accumulation in the extracellular space, which may result in multiple organ dysfunction [74,75].

### 3.3. TNF-α

TNF-α is also involved in the pathogenesis of many autoimmune disorders, including Ps and SLE. However, it has dual biological function. It has been found to have pro-apoptotic and pro-inflammatory activity at high concentrations but anti-apoptotic and anti-inflammatory activity at low concentrations [12,26,76,77,78]. It stimulates the proliferation and differentiation of T and B lymphocytes and increases the number of dendritic cells [12,79]. It is both a regulator of apoptosis and one of the most important pro-inflammatory cytokines; it upregulates the production of a range of pro-inflammatory molecules including CRP, prostaglandins, leukotrienes, platelet-activating factors, nitric oxide, and reactive oxygen species [80]. By increasing the synthesis of IL-6 and IL-1, it contributes to the activation of lymphocytes [79,81]. While TNF-α expression has been found to be elevated in the upper layers of the epidermis in both Ps and SLE patients [12,78,82,83,84,85], its involvement in the pathogenesis of these disorders remains unclear and controversial [12,78,86]. 

In Ps, TNF-α stimulates the proliferation and differentiation of both CD4+ and CD8+ T cells. It also contributes to the increased production of IL-17 by Th17 lymphocytes and IL-22 by Th1, Th17, and Th22 lymphocytes. IL-17 and IL-22 activate keratinocytes and enhance their proliferation. Moreover, IL-17 stimulates keratinocytes to produce TNF-α and the chemokine CCL20, which increase neutrophil migration into the affected epidermis [71,87].

## 4. Cytokines in SLE

SLE is a complex heterogenous disease with various cytokines involved in its pathogenesis; of these, IFN-α appears to play the most important role in the pathogenesis of SLE. Studies have found IFN-α to stimulate production of IL-17, and an exaggerated Th17 response has been noted in lupus patients together with elevated IL-17 sera levels. Although IL-17 and IL-23 inhibition was found to be effective in treating Ps, Th-17 targeted therapy did not demonstrate any therapeutic potential in lupus patients [88].

### 4.1. IL-17/IL-23/TNF-α

Studies indicate that the IL-17/IL-23/TNF-α axis activity plays a key role in the development of the inflammatory response and secondary organ damage in SLE [88]. IL-17 promotes the differentiation of B lymphocytes, and increased levels of IL-17 and IL-23 have been demonstrated in renal parenchymal biopsy specimens from patients with lupus nephritis compared to healthy subjects [89,90]. The importance of IL-17 in the pathogenesis of SLE has been confirmed by various other studies involving humans and animals [91,92,93,94]. Wong et al. report a significant increase in IL-17 levels and Th17 lymphocyte number in the plasma of SLE patients compared to healthy volunteers [91], and Tang et al. indicate a positive correlation between IL-17 level in blood and the severity of the disease course based on the SLEDAI scale [95]. 

Elevated serum IL-17A levels have been noted in patients with different types of lupus, such as SLE, discoid lupus erythematosus (DLE), and subacute cutaneous lupus erythematosus (SCLE). Also, a significant correlation was found between the number of Th17 lymphocytes in SCLE-type skin lesions and serum anti-Ro antibody titers [95,96,97,98]. Similar correlations were also observed among SLE patients with coexisting Ps [39,44]. A study using a mouse model of lupus with a genetic defect that blocks IL-17 production by Amarilyo et al. showed that ANA was not present in circulation in animals, and anti-dsDNA, anti-single-stranded DNA (anti-ssDNA), anti-ribonucleoprotein (RNP) and anti-chromatin antibodies were also not present [94]. 

Data suggest that IL-17 has a pathogenic role in the development of SLE. Indeed, the therapeutic use of anti-IL-17 inhibitors has been found to inhibit the clinical activity of SLE in the human population based on the SLEDAI scale. IL-17 antagonists are commonly used to treat moderate to severe Ps due to their efficacy [98]; however, there is no recognized treatment strategy for anti-IL-17 drugs in SLE. While these drugs have been found to be effective in a mouse model of lupus, human studies are required to establish their long-term safety and efficacy in SLE [97].

### 4.2. IL-2

IL-2 deficiency has been observed in various autoimmune diseases, including type 1 diabetes, RA, and SLE [98,99,100]; however, the mechanism explaining these reduced IL-2 levels is not fully understood. Reduced levels of IL-2 have been noted in the blood of SLE patients, and this has been associated with impaired transcription of the appropriate genes in T lymphocytes [101,102,103]. In addition, the level may be decreased by IL-23, the levels of which has been shown to be elevated in the plasma of SLE patients compared to controls. Higher IL-23 levels have also been correlated with SLE activity [104,105]. IL-2 Corrected as indicatedinhibits the differentiation of follicular T cells, the main function of which is to promote the proliferation and differentiation of B lymphocytes. Decreased IL-2 levels in SLE patients lead to elevated production of autoantibodies by B lymphocytes [106,107]. 

In SLE, the physiological role of the Treg/IL-2 axis is impaired [108]. The efficacy and good safety profile of low-dose IL-2 (aldesleukin) has been confirmed in a single-center clinical trial initiated in 2014; the study group comprised 12 patients with active SLE not responding to treatment with at least two classic drugs, such as corticosteroids, antimalarials, azathioprine, or mycophenolate mofetil [109]. IL-2 was administered subcutaneously daily in five-day cycles with a subsequent nine-day wash-out phase between cycles one and two, and a 16-days phase between cycles two and three, and cycles four and five. The dose of IL-2 in cycle one was 1.5 million IU and was increased by 1.5 million IU in each subsequent cycle. The results showed that in addition to selectively increasing the number of Tregs, IL-2 injections also reduced the number of CD19+ B lymphocytes. In 10 out of 12 patients (83%), disease activity decreased as assessed by the Safety of Estrogens in Lupus National Assessment-Systemic Lupus Erythematosus Disease Activity Index (SELENA-SLEDAI) scale; in addition, no severe exacerbations were noted during treatment. A reduction in SLE activity correlated with an increase in Treg counts. However, a modification of IL-2 dosing based on five-day cycles separated by a nine- to sixteen-day interval was associated only with a transient increase in Treg counts [109]. Another randomized, placebo-controlled study found low-dose IL-2 to elicit a reduction in SLE activity according to the SLEDAI score, which was also associated with a reduction in anti-dsDNA antibody titers [110]. 

### 4.3. TNF-α 

At high concentrations, TNF-α induces cell apoptosis. This may lead to exposure to autoantigens, production of autoantibodies, and, eventually, to the development of an autoimmune process [26]. A study on a group of 204 Indian female SLE patients by Mahto et al. found that the presence of the TNF-α gene polymorphisms G-238A and G-308A on chromosome 6 increase the risk of developing SLE compared to controls [111]. However, the large number of reports examining TNF-α levels in the serum of SLE patients have yielded contradictory results, with some reporting elevated TNF-α levels in the active phase of SLE and others indicating higher levels [81,112,113,114]. 

While the inhibition of TNFα production theoretically may be used to treat both SLE and Ps, reports indicate that therapeutic use exacerbated SLE symptoms. In addition, it has been shown that approximately 0.5–1% of patients receiving anti-TNFα treatment will develop lupus-like syndrome and 2–5% will develop paradoxical psoriasis [115,116,117,118,119,120]. Moreover, increased titers of anti-dsDNA and cardiolipin antibodies have also been observed in those treated with anti-TNFα [12]. 

## 5. Angiogenesis-Dependent Pathogenesis 

Pathological processes can be indicated by the presence of angiogenesis, i.e., the process of forming new blood vessels on the basis of pre-existing vessels. Under physiological conditions, it occurs sporadically during wound healing, endometrial regeneration during the menstrual cycle, and embryogenesis. However, it also plays an important role in the development of various pro- or anti-angiogenic diseases. In any case, the process is believed to be influenced by a number of factors. Increased neovascularization is a hallmark of many chronic inflammatory, autoimmune, and neoplastic processes, including SLE and Ps [121]. Ps is characterized by a number of pro-inflammatory factors such as increased numbers of blood vessels in the papillae of the dermis, their elongation, and enhanced permeability [121]. In SLE, angiogenesis can be enhanced by the deposition of immune complexes in the wall of blood vessels in various organs, including the skin; these damage the vascular endothelium and thus stimulate vessel growth [122].

### 5.1. Vascular-Endothelial Growth Factor 

Among the most potent stimulators of angiogenesis is vascular-endothelial growth factor (VEGF). Its concentration is elevated in both Ps and SLE compared to the general population [123]. While IL-17 plays an important pro-inflammatory role in both diseases, it has also been shown to stimulate vascular proliferation by directly increasing the release VEGF and IL-8, a potent chemotactic and angiogenic chemokine [124]. Skin biopsy specimens obtained from active psoriatic lesions have indicated higher expression of VEGF-A compared to healthy skin of Ps patients and the skin of healthy people [121]. In addition, plasma VEGF levels have been found to be elevated in Ps patients compared to healthy subjects, with these levels correlating positively with disease severity, as assessed by the PASI scale [125,126,127]. 

Psoriatic lesions have been found to harbor elevated levels of TNF-α produced by lymphocytes and keratinocytes. TNF-α increases the expression of VEGF-R on the surface of endothelial cells, thereby promoting angiogenesis [121]. Similarly, in a study of 75 SLE patients, Liu et al. found plasma VEGF levels to be significantly higher in subjects with active SLE (SLEDAI > 4) compared to healthy controls and patients with inactive SLE (SLEDAI ≤ 4). The association between plasma VEGF levels and disease activity has also been repeatedly confirmed in other studies [128,129,130,131,132,133]. Moreover, studies have found higher plasma VEGF levels in SLE patients with renal involvement, with the levels correlating with the severity of renal impairment [123,134]. 

Interestingly, both the gene encoding VEGF and the susceptibility genes for Ps and SLE are located on the short arm of chromosome 6 [135,136,137,138,139]. In Ps, the best known susceptibility locus is PSORS1, whereas in SLE, the most significant are the HLA class II (HLA-DR2 and HLA-DR3) and class III (MSH5 and SKIV2L) gene polymorphisms. All of the aforementioned loci are located in the 6p21.3 region [135,140,141,142,143,144,145,146]. 

Patients with SLE have also been found to demonstrate elevated serum TNF-α levels compared to controls [123]. TNF-α has also been shown to induce an increase in the expression of receptors for various proangiogenic factors, such as VEGF, IL-8, basic fibroblast growth factor (bFGF), angiopoietin (Ang), and Tie-2 receptor [147].

### 5.2. Angiopoietins 

Another group of proangiogenic cytokines that have been analyzed in the pathogenesis of various diseases is the angiopoietins (Angs), comprising Ang-1 to Ang-4; of these Ang-1 and Ang-2 are crucial in angiogenesis. Upon binding to the endothelial tyrosine kinase receptor, known as Tie2, Ang-1 initiates the processes responsible for vascular maturation and stability, ensuring endothelial cell homeostasis [121]. In contrast, Ang-2 as a competitive antagonist of the Tie2 receptor promotes instability, increased permeability, and structural abnormalities in the blood vessels [148]. Among the Angs, Ang-2 plays a leading role in the pathogenesis of vascular abnormalities in both Ps and SLE, in which the cytokine acts synergistically with VEGF-A to stimulate neovascularization. The Ang/Tie2 complex, in association with VEGF-A, has been shown to co-initiate vascular proliferation in the psoriatic plaque [127,149]. 

Angiopoietins are believed to be involved in the pathogenesis of Ps, as indicated by a study by Koroda et al. The research was designed to assess whether improvement in psoriatic lesions is associated with changes in the expression of Angs and Tie2. Thus, seven patients who received effective antipsoriatic therapy, five patients treated with PUVA therapy, and two patients treated with topical tazarotene were included in the study group [150]. After eight weeks of treatment, visible clinical improvement was achieved in each subject. Histopathological findings demonstrated a significant reduction in Ang-1, Ang-2, and Tie-2 levels in the involved skin following treatment compared to baseline [150]. 

Similarly, in SLE, elevated plasma Ang-2 levels were reported compared to healthy volunteers, with the concentration correlating positively with SLE activity and the risk of lupus nephritis [151]. Negative correlations with C3 and C4 concentrations and with eGFR and flow mediated dilatation (FMD) were also reported. Ang-2 has been shown to increase the sensitivity of endothelial cells to the pro-inflammatory effects of TNF-α, contributing to vascular inflammation and facilitating the formation of an inflammatory infiltrate by neutrophils and monocytes [151].

### 5.3. Fibroblast Growth Factor 

The fibroblast growth factor (FGF) family also plays an important role in angiogenesis. Several studies have found plasma FGF-23 levels to be elevated in SLE patients, particularly in cases of lupus nephritis. However, a study of 60 patients with SLE found no significant difference in plasma FGF-23 levels between patients with renal involvement and those without (106.49 ± 88.2 vs. 93.84 ± 74.67 pg/mL, *p* = 0.56), and similar plasma FGF-23 levels were noted in SLE patients and healthy controls (99.6 ± 79.86 vs. 139 ± 12.3 pg/mL, *p* > 0.05). Hence, further studies are recommended to better understand these mechanisms [152]. 

Studies have also noted higher FGF-23 concentrations in Ps patients compared to healthy subjects, as well as an association between elevated FGF-23 concentrations and a higher prevalence of insulin resistance, dyslipidemia, and atherosclerosis [153,154,155,156]. 

## 6. Clinical Features 

SLE and Ps are both incurable autoimmune diseases characterized by a chronic and relapsing course. Data from the literature indicate that the prevalence of SLE in the course of Ps is 0.69%, while Ps is detected with a frequency of 1.1% in patients previously diagnosed with SLE [20]. The literature also suggests that Ps is usually detected before SLE. Millns et al. reported that Ps precedes the first symptoms of SLE by at least five years; in cases when SLE is confirmed earlier, the diagnosis of Ps is made approximately 2.2 years later [9]. Establishing a correct diagnosis based on the clinical presentation of skin lesions is often difficult, as they may demonstrate similar morphology. Although these are two separate disease entities, each with their own characteristic clinical picture, diagnostic problems can still occur, especially in situations when they coexist. In these cases, further diagnostics are necessary to avoid errors in choosing the appropriate therapy [157].

Skin lesions in lupus can be subdivided into acute, subacute, and chronic forms [46]. Among the different subtypes of SLE, SCLE is characterized by a milder course. Its lesions also bear the closest clinical resemblance to psoriatic lesions; however, unlike Ps, SCLE is characterized by a marked sensitivity to UV radiation. Although arthritis is quite common in SCLE, lupus nephritis, neurological symptoms, and vasculitis are rare according to the literature, with an estimated frequency of around 10% [39]. SCLE can be observed as either psoriasiform or annular forms. In both, the skin lesions are most commonly located on the trunk (especially the upper back) and extensor parts of the arms and forearms, similar to Ps [158]. However, SCLE lesions can also appear on the lower extremities and the scalp, and in the case of psoriasiform lesions, they may be accompanied by the Koebner phenomenon, which is characteristic of the active phase of Ps [158,159].

The clinical picture in Ps is usually characteristic, with the lesions being located in typical areas; hence, diagnosis does not present any serious problems (Figure 2). The primary lesions present as inflammatory papules with scales on the surface, merging into erythematous and exfoliative foci, resembling psoriasiform lesions in SCLE. A key differentiating feature is the frequent involvement of the nail plates and genital areas in Ps and the lack of such in SCLE. In cases where the skin lesions do not affect these locations, the differential diagnosis should be based on the histopathological picture. Regardless of the clinical evidence, these histopathological findings are also key to distinguishing the two diseases [160]. 

**Figure 4:**
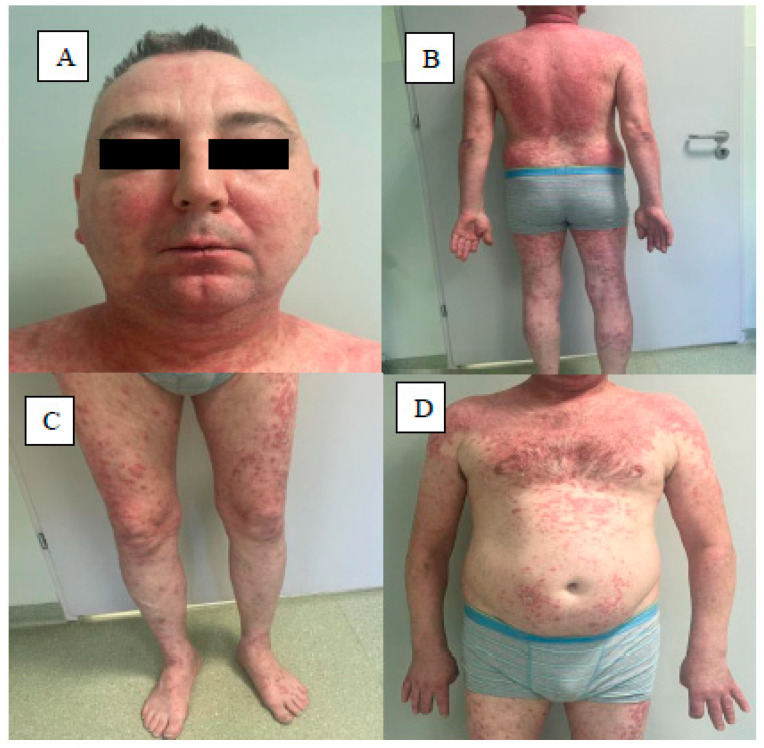
Patient with coexisting psoriasis and SLE. Facial lesions of erythema covering the cheeks and bridge of the nose with a silver lip symptom in the course of SLE (**A**). Psoriatic lesions, erythematous papules covered with silvery scales, on the trunk and upper and lower limbs (**B**,**C**). Psoriasis-like lesions in the upper trunk typical of SCLE (**D**).

There are few reports in the available literature describing cases of coexistence of both diseases, and little information exists regarding the clinical picture of skin lesions in such cases (Figure 4). Millns et al. report that patients first diagnosed with SLE present with skin lesions typical of Ps, such as papules and erythemato-exfoliating foci, in areas exposed to UV radiation. This picture may indicate photoprovocation of Ps in a patient with SLE, in whom UV hypersensitivity is one of the primary symptoms of lupus. In typical Ps, UV radiation is used in the therapy of the disease and rarely provokes its exacerbation. Patients with both diseases typically demonstrate high sensitivity to UV radiation, much more so than when these diseases occur alone. Millns et al. observed photosensitivity in 23 out of 27 patients diagnosed with SLE and Ps (85.2%) [160]. 

Data from the literature indicate that UV hypersensitivity occurs in 5.5% of patients with chronic Ps [161] and around 60% of patients with SLE, i.e., 63% in SCLE and 45% in DLE [162]. Tselios et al. report no statistically significant difference in the frequency of SLE exacerbations before and after the diagnosis of Ps, based on an analysis of 63 cases of Ps. In addition, Ps did not appear to have any influence on increased medical burden in patients with SLE, manifested as the incidence of cardiovascular events and venous thromboembolic disease [16]. It is common that an exacerbation of one disease is associated with a worsening of the other. The typical basal lesions of Ps, i.e., papules covered with silvery scales, can be observed in both diseases, and only thorough diagnostics, including a careful analysis of the clinical picture supported by the result of the histopathological examination, can provide a definitive diagnosis [9].

Arthropathy can also pose diagnostic difficulties in cases where the coexistence of Ps and SLE is suspected. Psoriatic arthritis (PsA) manifests as seronegative inflammation with a heterogeneous spectrum of symptoms that can evolve over the course of the disease, eventually leading to severe and irreversible damage and disability. PsA can affect peripheral joints and the spine and can precede the onset of skin lesions, complicating the diagnosis of Ps. Patients with PsA tend to be characterized by a mild severity of skin lesions. Joint involvement is common in both Ps and SLE. Diagnosis of PsA is usually based on the Classification Criteria for Psoriatic Arthritis (CASPAR), imaging studies, and laboratory tests. The prevalence of PsA in the group of patients with SLE and Ps is significantly higher than in patients with only Ps, reaching 4.5% [163].

Arthritis is very common in SLE, occurring even up to 95% of patients in some studies. It is a seronegative condition but characterized by a variety of symptoms characteristic of various types of arthropathy in the course of SLE. It is often accompanied by other symptoms, such as general weakness and myalgia. The variety of forms and severity of symptoms can cause diagnostic difficulties, similar to those seen in Ps [164].

Both Ps and SLE can contribute to kidney damage. Although lupus nephropathy is more commonly described in the literature, Ps has also been associated with the risk of kidney damage that may lead to chronic kidney disease requiring dialysis. The term psoriatic nephropathy encompasses a number of forms of nephritis associated with Ps, including membranous glomerulonephritis, IgA nephropathy, and mesangial glomerulonephritis [165,166,167]. Proteinuria as a manifestation of renal disease is increasingly common in patients with Ps, especially in those with diagnosed PsA [168]. There are also case reports suggesting that hemodialysis therapy is a risk factor for the development of Ps. No similar risk is observed in PsA [169]. 

Renal involvement is also a common manifestation of SLE and is therefore included in the classification criteria [23]. Indeed, kidney disease is a major risk factor in the course of SLE. Histopathology indicates six classes of lupus glomerulopathy: class I with minimal changes, class II with proliferative changes in the mesangium accompanied by deposits, class III with focal proliferative changes in the glomeruli, class IV with extensive proliferative changes in the glomeruli involving at least 50% of the glomeruli, class V membranous glomerulonephritis, and class VI advanced glomerular sclerosis. This classification has significance for diagnosis, treatment, and prognosis [170].

The key tool for differentiating between certain disease entities with similar clinical presentations is histopathological examination. Cutaneous lupus erythematosus is characterized by interface dermatitis with lymphocytic infiltration and keratinocyte necroptosis at the dermo-epidermal junction [171]. A psoriatic plaque usually includes parakeratosis, Munro’s microabscesses formed by neutrophils migrating from the dermis to the epidermis, and elongation of the dermal papillae together with minor swelling of the papillary dermis. Ps on the other hand is characterized by tortuous dilated vessels, highlighting the important contribution of angiogenesis. It can be seen that SLE and Ps demonstrate characteristic histopathological features, allowing an accurate diagnosis to be made [172].

## 7. Treatment 

Different local and systemic therapeutic approaches are used in the treatment of Ps and SLE, which can pose many problems (Table 1). In cases where both Ps and SLE coexist, treatment is more likely to be unsuccessful. There are numerous cases described in which SLE treatment paradoxically induced Ps and, conversely, Ps therapy led to the manifestation of SLE or the development of lupus-like syndrome. For example, the UVA and UVB phototherapy commonly used to treat Ps induces or exacerbates SLE symptoms; as such, photoprotection is recommended for all patients [9,49,173]. Conversely, antimalarials, such as hydroxychloroquine and chloroquine, the gold standard in SLE treatment, may exacerbate concomitant Ps or trigger drug-induced psoriasis-like lesions [12,39,174]. 

An effective systemic therapeutic agent for both Ps and SLE is the folic acid analogue methotrexate (MTX). MTX inhibits dihydrofolate reductase, reducing the synthesis of pyrimidines and purines and ultimately inhibiting the division of rapidly dividing cells, such as keratinocytes and bone marrow cells. In addition to its antiproliferative effect, MTX has anti-inflammatory and immunosuppressive activity (Table 1) [175,176]. MTX is an effective drug in moderate to severe plaque Ps, as well as in other forms of Ps, such as erythroderma psoriasis, generalized pustular psoriasis, nail psoriasis, palmoplantar Ps, and PsA [176]. In Ps, MTX not only normalizes keratinocyte proliferation, but also prevents the migration of inflammatory cells into the skin by reducing the levels of the endothelial adhesion proteins ICAM1 and E-selectin. In addition, the drug inhibits angiogenesis, interferes with antigen presentation by dendritic cells, and reduces the number of autoreactive T lymphocytes expressing cutaneous lymphocyte antigen (CLA); it also has a cytotoxic effect on T lymphocytes mediated by free oxygen radicals and reduces plasma TNF-α levels. These processes and mechanisms are key pathogenetic elements in both Ps and SLE. Clinically, the drug’s efficacy in arthritis makes it the first choice for joint involvement in both diseases [177]. 

Fan et al. showed that MTX reduced tissue infiltration by TNF-a, IL-6, and IL-23 in mice with collagen-induced arthritis (CIA); it also inhibited B-lymphocyte differentiation, restored the balance between the regulatory B cells (Bregs) and dendritic cell populations, and enhanced the production of the anti-inflammatory cytokine IL-10 [177]. MTX also contributes to an increase in complement components C3 and C4 and a decrease in antibody titers, including anti-dsDNA. MTX can therefore be used successfully in Ps and SLE, despite the diseases having quite different cellular pathways [178]. 

A classical immunosuppressive drug used to treat both diseases is the calcineurin inhibitor CsA. Although this drug shows nephrotoxicity, it is approved for the treatment of lupus nephritis during the period of proper renal function. CsA is preferred in young and middle-aged patients with stabilized blood pressure. Calcineurin inhibitors suppress IL-2 production, representing a well-established treatment for various immune diseases. The agents also inhibit T-lymphocyte proliferation and the production of pro-inflammatory cytokines, including IL-1, IL-2, IL-3, IL-4, IL-5, GM-CSF, TNF-α, and IFN-γ

As Ps and SLE have an immune and inflammatory background, calcineurin inhibitors have been used in both diseases. Although CsA is one of the classic systemic treatments for both diseases, tacrolimus is also used off-label in Ps therapy as an effective form of topical treatment for sensitive areas such as the face, genitals, and excoriation regions [179].

Recently biologic drugs have aroused considerable interest as treatments. They are believed to offer greater efficacy, less frequent dosing, and less toxicity than conventional treatments. The drugs are proteins that act selectively on specific elements of the immune system and thus have a good safety profile and limited side effects when used in targeted therapy. While certain drugs are known to be effective for both SLE and Ps alone, their effectiveness against the two together remains unknown [180].

The anti-CD20 monoclonal antibody rituximab has been evaluated for the treatment of SLE and Ps. The CD20 molecule is expressed on the surface of B lymphocytes, and its blockage inhibits B-cell proliferation and activation, thus suppressing autoantibody production [12]. Rituximab reduces plasma levels of the pro-inflammatory cytokine IL-22 and decreases the number of activated Th17 lymphocytes. Hence, it appears that it may be an alternative therapeutic option for the treatment of concurrent SLE and Ps in cases where other therapies have failed [12,180]. 

However, few reports have described the clinical improvement of psoriatic skin lesions and joint complaints in PsA after treatment with rituximab [181,182,183]. Chang et al. present a case report of a woman with palmoplantar psoriasis (PPP), in whom phototherapy, topical corticosteroids, leflunomide, MTX, CsA, and etanercept treatment was ineffective; in this case, rituximab administration resulted in a six-month clinical improvement [181]. When commonly employed treatments are ineffective, rituximab can be included. Rituximab treatment was also found to induce an improvement in SLE patients, expressed as a reduction in disease activity according to the SLEDAI-2K scale after only three months. Treatment was also associated with a reduced risk of subsequent exacerbations. The mean time from initiation of therapy to complete remission was 20 months. In addition, treatment yielded a reduction in anti-dsDNA titers, an increase in platelet counts, but no significant difference in C3 and C4 concentrations [184]. 

As rituximab is a potent inhibitor of B-lymphocyte activity, it is used in the management of a wide variety of diseases, including autoimmune diseases and certain cancers. As SLE and Ps are both autoimmune disorders, the inclusion of anti-CD20 drugs seems reasonable; however, the efficacy of anti-CD20 drugs in both diseases is not confirmed. In addition, some studies report that the therapeutic use of anti-CD20 drugs in SLE patients induced the development of Ps or psoriasis-like lesions [12,185,186,187,188,189]. 

Currently, a wide group of biological drugs with good safety profiles have been proven to be effective in Ps therapy [190]; however, such regimens are not standard methods for treating SLE. Some biologics have been used in the treatment of SLE, including anifrolumab, a human monoclonal antibody directed at subunit 1 of the type 1 interferon receptor, and belimumab, an inhibitor of B-cell stimulator protein; however, these are not recommended for treating Ps due to the lack of a satisfactory therapeutic effect or a lack of confirmation in clinical trials [191,192,193,194].

Hence, in cases where both conditions are present, particular care should be taken when choosing a biological drug. There is therefore a need to conduct further research aimed at explaining the exact pathogenetic mechanisms of both these diseases [9]. A comparison of treatment methods for both diseases is presented in Table 1.

## 8. Conclusions

PS and SLE are autoimmune diseases in which chronic inflammation is a key factor in their pathogenesis. Despite presenting seemingly different etiopathogeneses, in both cases, inflammatory mechanisms are stimulated by the same immune cells and inflammatory cytokines. Ps and SLE rarely co-occur, and such cases can pose numerous diagnostic and therapeutic challenges. As standard treatment methods for each of these diseases may induce lupus-like syndrome or psoriasis-like skin lesions, current research is focused on developing therapeutic strategies targeting the shared pathogenetic pathways of both diseases.

## Figures and Tables

**Table 1:** Comparison of treatment methods in SLE and Ps.

Systemic Lupus Erythematosus	Psoriasis
Topical treatment	
Glucocorticosteroids	Glucocorticosteroids
Calcineurin inhibitors (tacrolimus, pimecrolimus)	Calcineurin inhibitors (tacrolimus, pimecrolimus)
	Vitamin D derivatives (calcipotriol, tacalcitol)
	Agents with salicylic acid or urea
	Tar preparations
	Cygnoline
Phototherapy	
Not applicable, photoprotection recommended	UVA-PUVA, UVB-NB
Systemic treatment	
Glucocorticosteroids	Glucocorticosteroids
Methotrexate Cyclosporine A Mycofenolate mofetil Azathioprine Antimalarial drugs (hydroxychloroquine, chloroquine)	Methotrexate Cyclosporine A Mycofenolate mofetil Acitretin
Biological treatment	
Human monoclonal antibody to the type 1 interferon receptor (anifrolumab) Human monoclonal antibody that inhibits B-cell activating factor (belimumab)	Blockers of tumor necrosis factor-α Interleukin 12 and 23 (IL-12/23) inhibitors IL-17 inhibitors IL-23 inhibitors

## Data Availability

Data sharing is not applicable.
